# Editorial: Public health strategies to improve mental health in the education sector: perspectives and applications

**DOI:** 10.3389/fpubh.2026.1909208

**Published:** 2026-07-07

**Authors:** Diana Patricia Guizar-Sánchez, Ana Fresán-Orellana, Raúl Sampieri-Cabrera

**Affiliations:** 1 Department of Physiology, Faculty of Medicine, National Autonomous University of Mexico, Mexico City, Mexico; 2 Instituto Nacional de Psiquiatria Ramon de la Fuente Muniz, Mexico City, Mexico; 3 Centro de Ciencias de la Complejidad, Universidad Nacional Autónoma de México, Mexico City, Mexico

**Keywords:** complex adaptive systems, educational mental health, implementation science, mental health literacy, psychological safety, public health, whole school approaches

## Introduction

The mental health of students and educators has moved from the margins of educational policy to the center of public health debate (1, 2). This shift reflects more than just an increase in diagnostic awareness in the field. This signals a deeper transformation in the conditions under which young people learn, teachers work, and institutions govern their care (3). Before the COVID-19 pandemic, mental disorders were a major cause of disability among children, adolescents, and young adults (4). Global burden analyses estimate that hundreds of millions of people aged 5–24 live with at least one mental disorder, with anxiety, depression, self-harm, substance use, and eating disorders concentrated during periods of educational transition (5, 6). The pandemic intensified this trajectory by disrupting peer connections, family stability, sleep, physical activity, access to care and institutional trust (7).

However, the dominant policy response is structurally conservative. Most educational systems continue to operate through a late-stage, individualized model: distress is detected once it becomes visible, students are referred to scarce services, responsibility is transferred to counselors or clinicians, and institutional determinants remain underexamined (8). This model is important but insufficient. It treats education as a venue for mental health services rather than as a determinant of mental health.

The central claim of this editorial is that mental health in education should be understood as an emergent property of complex adaptive systems (CAS). Psychological distress does arise within individuals, but it is patterned by learning environments, academic norms, belonging, humiliation, digital exposure, precarity, discrimination, workload, teacher burnout, institutional responsiveness, and the availability of credible support pathways. Therefore, the public health task is broader than scaling up counseling provisions. This requires the redesigning of educational ecosystems.

## From individual distress to educational ecosystems

The biomedical model provides indispensable tools for diagnosis, treatment, and risk management. Its limitations appear when it becomes the only grammar available for interpreting educational distress. A student who develops anxiety in a highly competitive school, a medical trainee who withdraws after repeated humiliation, or a teacher who experiences burnout under impossible workload conditions cannot be adequately understood through individual vulnerability alone. Their suffering is located within a network of institutional arrangements.

Ecological approaches shift the unit of analysis. They ask how classrooms, curricula, assessment regimes, peer hierarchies, digital infrastructures, and leadership practices produce mental health exposure. This move aligns with the social determinants of mental health framework, which links psychological outcomes to poverty, housing, discrimination, family stress, violence, migration, social isolation, and access to care (9, 10). In educational systems, these determinants operate through daily institutional mechanisms such as tracking practices, punitive discipline, competitive grading, exclusionary cultures, unstable accommodation, food insecurity, and limited access to trusted adults.

Whole-school approaches have emerged from this recognition. Their premise is that mental health promotion requires coordinated change across the curriculum, staff development, student participation, leadership, family engagement, safeguarding, anti-bullying policy, and referral pathways (11). The evidence suggests promise, especially when interventions alter the wider school climate rather than deliver isolated psychoeducational sessions (12). Simultaneously, effect sizes are heterogeneous, long-term outcomes remain uneven, and implementation quality strongly conditions the impact (13). This should temper enthusiasm. Whole-school approaches are better understood as governance frameworks rather than as discrete interventions.

Psychological safety is central to this ecosystemic approach. Originally developed in organizational studies and increasingly applied to health professions education, psychological safety refers to the perceived possibility of speaking, questioning, erring, and seeking help without humiliation or punishment (14). In educational settings, psychological safety links learning and mental health. Environments that punish uncertainty generate silence; silence conceals distress, weakens help-seeking, and impoverishes learning. In contrast, psychologically safe environments allow students and staff to disclose difficulties early, repair mistakes, and sustain epistemic risks (15).

Belonging operates through related pathways. Reviews and longitudinal studies have associated school belonging with lower depressive symptoms, stronger engagement, and improved wellbeing (16). However, a sense of belonging should not be reduced to emotional warmth. It is a structural indicator: students feel they belong when institutions recognize, protect, include them in decision-making, and make success plausible across different social groups. Therefore, belonging is a public health metric and an educational aspiration (17).

## Mental health as a public health and educational governance challenge

The mental health crisis in education is fundamentally a governance crisis. Institutions continue to implement mindfulness workshops, awareness campaigns, resilience training, screening initiatives, digital applications and referral systems. Although many of these efforts are well-intentioned, they rarely alter the organizational conditions that repeatedly produce psychological distress. The result is a cycle of highly visible but poorly integrated interventions that often achieve limited reach, inconsistent implementation, weak sustainability, and little influence on institutional decision-making (18).

The governance approach asks different questions than the traditional approach. Who is accountable for mental health in institutions? What indicators were monitored? How are the voices of the students and staff incorporated? How do the health, education, and community sectors coordinate the care? Which practices require de-implementation because they are harmful? Implementation science has become indispensable in this context. Recent work in school mental health emphasizes pragmatic implementation strategies, mechanisms linking strategies to outcomes, intervention redesign for real-world settings, and de-implementation of low-value practices (19, 20).

Educational governance must also move across different levels. At the macro level, ministries and accreditation bodies can embed mental health into quality assurance, school inspections, teacher education, and higher education regulations. At the meso level, institutions can create multidisciplinary governance units that integrate student affairs, academic leadership, health services, safeguarding, data systems and community partners. At the micro level, classrooms can adopt relational pedagogies, fair assessment, inclusive participation, and early support practices (21, 22).

Public health framing matters because it changes ethical horizons. Clinical services target cases, and public health systems reduce incidence, severity, and inequity. In education, this implies upstream action on academic pressure, bullying, discrimination, sleep disruption, financial stress, loneliness and digital harm (1). It also requires the attention of teachers and staff members. Educators are often treated as delivery agents for student wellbeing, while their own psychological burdens remain institutionally neglected. A sustainable educational system cannot rely on exhausted adults to protect vulnerable students (23).

## Prevention, early intervention, and mental health literacy

The strongest argument for schools and universities as mental health settings is prevention. Most mental disorders begin before or during the years in which people pass through educational systems. Therefore, it offers a platform for universal prevention, selective support, and early intervention. The evidence supports a cautiously optimistic approach to this Research Topic. School-based mental health literacy interventions can improve knowledge, reduce stigma, and support help-seeking attitudes (3, 24). Universal and targeted school-based interventions for depression and anxiety show benefits, although the effects vary by design, age, delivery, follow-up, and socioeconomic settings (25). Whole-school interventions appear promising, but their impact depends on fidelity, leadership, and the depth of organizational change (26, 27).

Mental health literacy should be understood as more than just information on symptoms. A mature literacy model includes recognition of distress, knowledge of care pathways, stigma reduction, peer support boundaries, digital risk awareness, crisis response, sleep and activity literacy, and the capacity to distinguish normal distress from clinically relevant impairment. For teachers, literacy also includes the ability to notice patterns without becoming a substitute for clinicians. For institutions, literacy means knowing how organizational routines produce mental health outcomes.

Early intervention also faces similar challenges. Identifying distress is of limited value when timely and appropriate support is unavailable. Screening programs that operate without adequate service capacity risk creating unmet expectations, while digital assessment systems implemented without robust privacy safeguards can undermine trust. Similarly, referral mechanisms are unlikely to reduce disparities if the available services fail to meet the needs of diverse populations. The effectiveness of early intervention depends not only on detection but also on the strength of the entire support pathway, from assessment and triage to treatment, follow-up, and continuous evaluation.

The prevention agenda also requires this humility. Resilience language can easily become institutional evasion: students are trained to adapt to harmful environments rather than institutions being required to reduce avoidable harm. Educational resilience refers to the adaptive capacity of the entire system rather than merely individual endurance. A resilient educational system learns from distress signals, redistributes support, revises harmful policies, and protects the relational conditions of learning.

## Digital transformation, artificial intelligence, and the future of educational mental health

Digital technologies are reshaping educational mental health in contradictory ways. They create opportunities for scalable psychoeducation, low-threshold support, ecological monitoring, tele-mental health, peer navigation and stepped-care systems. Reviews of digital mental health interventions among university students suggest potential benefits, particularly when the interventions are evidence-based, guided, integrated into services, and designed for sustained engagement (28). School-based e-health interventions may also support wellbeing, although the evidence base is still being developed (29).

Digital life is simultaneously part of the problem architecture. Cyberbullying, social comparison, sleep displacement, algorithmic amplification, online harassment, and compulsive platform use interact with anxiety, depression, and self-harm risks (30). Universities and schools can no longer treat the digital sphere as external to education. For many students, the learning environment extends to messaging platforms, social media, learning management systems, and AI-mediated communication (31, 32).

Artificial intelligence intensifies both promise and risk. AI systems may support early warning, triage, personalized psychoeducation, administrative burden reduction, and surveillance at the population level. Generative AI may also offer conversational support, translation, simulation, and mental health literacy. However, recent reviews have emphasized ethical concerns regarding accuracy, bias, privacy, accountability, safety, transparency, and clinical overreach (33). In educational settings, these risks become sharper because users are often minors or young adults embedded in power relations with institutions.

Therefore, the future of digital educational mental health should be governed by strict principles: data minimization, informed participation, algorithmic audits, human oversight, equity analysis, crisis escalation protocols, and separation between supportive monitoring and disciplinary surveillance. Institutional trust is a critical variable. Students and staff will only participate in digital mental health systems if they believe that the data will be used to support rather than punish them.

## Toward a new research agenda

A new research agenda must move beyond the question, “Which program works?” The more relevant question is: “Which combinations of institutional conditions, intervention components, implementation strategies and social protections improve population mental health, for whom, under what circumstances and at what cost?”

First, longitudinal designs are required to address this Research Topic. Cross-sectional surveys describe the burden of disease, but cannot adequately capture developmental timing, institutional exposure, causal pathways, or delayed effects. Educational mental health research should follow cohorts across transitions, such as primary school to secondary school, secondary school to university, preclinical to clinical training, and graduation to employment.

Second, future research should incorporate the complexity of science. Schools and universities are complex adaptive systems in which interventions can produce nonlinear effects, feedback loops, and unintended consequences (34). Network science can map peer contagion, belonging, help-seeking pathways, bullying dynamics and institutional referral bottlenecks. System dynamics can be used to test how staffing, demand, waiting times, and crisis escalation interact with each other. Agent-based models can explore how policy changes alter the risk distribution across heterogeneous populations.

Third, mixed methods should be the standard rather than a supplement. Quantitative indicators can identify population patterns, whereas qualitative inquiry can explain how students and educators experience policy, stigma, trust, exclusion, and care. Participatory methods are especially important because institutional mental health strategies often fail when they are designed without the people who must use them.

Fourth, implementation science should be embedded in the beginning of the program. Trials should measure the acceptability, feasibility, fidelity, adaptation, cost, equity, and sustainability. Many interventions fail because they are too complex for real classrooms, poorly aligned with teacher workloads, or dependent on temporary funding. Pragmatic trials, hybrid effectiveness-implementation designs, and comparative studies of governance models are needed.

Fifth, new indicators are required. Educational systems usually measure attendance, grades, completion, retention and satisfaction. However, these indicators are insufficient for this purpose. Population mental health governance should include belonging, psychological safety, help-seeking confidence, staff wellbeing, perceived fairness, loneliness, exposure to bullying, sleep opportunities, digital harm, service accessibility, referral completion, and institutional trust.

## Conclusion

The next decade will determine whether educational mental health becomes another fragmented policy slogan or a genuine public health transformation. Evidence already supports a decisive shift: from individual distress to ecosystems, from programs to governance, from awareness to literacy, from crisis response to prevention, from digital enthusiasm to ethical infrastructure, and from short-term evaluation to adaptive learning systems.

Schools and universities are among the most powerful mental health infrastructures in society. They shape daily routines, identity, aspirations, relationships, and future possibilities. When they generate humiliation, exclusion, overload, and silence, they become risky environments. When they cultivate belonging, fairness, psychological safety, and credible care, they become the engines of human development.

Therefore, the task is political, scientific, and pedagogical in nature. Researchers must build multiscale models that match the complexity of these problems. Institutions must redesign learning conditions rather than outsourcing distress to under-resourced services. Policymakers must consider mental health education as a population-level investment in social capability. The future of mental health in education will not be secured by adding isolated interventions to unchanged educational systems. This will depend on whether educational systems acquire the courage and intelligence to transform themselves.
